# First-Trimester Maternal Serum Growth Arrest-Specific Protein 6 (Gas6) Levels for the Prediction of Preeclampsia

**DOI:** 10.3390/jcm15145604

**Published:** 2026-07-17

**Authors:** Zehra Yılmaz, Nazan Yurtcu, Dilay Karademir, Canan Soyer Calıskan, Samettin Celik

**Affiliations:** 1 Department of Obstetrics and Gynecology, Private Practice, 55200 Samsun, Türkiye; z.a.yilmaz@gmail.com; 2 Department of Obstetrics and Gynecology, Sivas Cumhuriyet University, 58140 Sivas, Türkiye; dr.dilaykarademir@gmail.com; 3 Department of Obstetrics and Gynecology, Samsun University Faculty of Medicine, Samsun Training and Research Hospital, 55090 Samsun, Türkiye; canansoyer@hotmail.com (C.S.C.); drsamettincelik97@gmail.com (S.C.)

**Keywords:** preeclampsia, growth arrest-specific protein 6 (Gas6), first-trimester screening, predictive biomarkers, neonatal outcome

## Abstract

**Objective**: Preeclampsia (PE) is a leading cause of maternal and perinatal morbidity; however, reliable first-trimester biomarkers are limited. Growth arrest-specific protein 6 (Gas6), a vitamin K-dependent ligand for the TAM receptor family (Tyro3, AXL, and MerTK), is crucial for trophoblast invasion and placental remodeling. This study evaluated whether first-trimester serum Gas6 levels differed between women with PE and controls and assessed the predictive utility across phenotypes. **Methods**: Participants were enrolled prospectively during routine first-trimester screening at 11 + 0 to 13 + 6 weeks, and cases and controls were selected using a nested case–control design after pregnancy outcomes were known. ROC-derived cut-off values for Gas6 were selected retrospectively using the Youden index and were considered exploratory. The PE group was divided into early (*n* = 15, <34 weeks) and late onset (*n* = 65, ≥34 weeks) subgroups. ELISA measured serum Gas6. Mann–Whitney U, Kruskal–Wallis, Spearman’s correlation, ROC analysis, and multivariate logistic regression were applied. **Results**: Gas6 levels were significantly lower in PE than controls (15.73 vs. 38.08 ng/mL, *p* < 0.001); control reference median (38.08 ng/mL, IQR 27.62 to 49.14), decreasing progressively to late (20.07 ng/mL) and early onset PE (6.45 ng/mL; all *p* < 0.001). ROC analysis showed the highest exploratory discrimination for early onset preeclampsia (AUC = 0.959, 95% CI: 0.923–0.995; sensitivity 93.3%, specificity 84.1% at a retrospectively selected cut-off of ≤13.82 ng/mL), with more modest discrimination for overall PE (AUC = 0.762) and late onset PE (AUC = 0.708). Lower first-trimester Gas6 levels remained significantly associated with early onset PE after adjustment for maternal age and BMI (OR = 0.555, 95% CI: 0.412–0.748, *p* < 0.001). **Conclusions**: Serum Gas6 levels in the first trimester are lower in PE, particularly in cases of early onset disease, highlighting its potential as a candidate biomarker that requires further prospective and external validation before any screening application can be considered.

## 1. Introduction

Preeclampsia (PE) is a complex, pregnancy-specific multisystem syndrome characterized by the de novo development of hypertension after the 20th week of gestation, typically accompanied by proteinuria or maternal end-organ damage [1,2]. Global estimates suggest that PE complicates approximately 2–8% of all pregnancies, contributing to more than 50,000 maternal deaths annually and maintaining its status as a leading driver of maternal and perinatal morbidity [3]. The clinical spectrum of PE is conventionally bifurcated into early (<34 weeks) and late onset (≥34 weeks) phenotypes, which are distinct entities that exhibit significant divergence in their underlying pathophysiology, clinical trajectory, and ultimate prognosis [4].

Early onset PE is primarily rooted in an aberration of deep placentation, in which the failure of trophoblastic infiltration hampers the physiological remodeling of spiral arteries, leading to placental ischemia and a subsequent systemic surge in anti-angiogenic mediators [5]. In contrast, the pathogenesis of late onset PE, while also involving placental factors, appears to be more heavily influenced by maternal constitutional vulnerabilities, such as obesity, metabolic syndrome, and pre-existing endothelial dysfunction [6]. From a prognostic perspective, the necessity of differentiating these two entities is paramount, given that early onset disease is associated with significantly more adverse maternal and neonatal outcomes [7].

To date, screening protocols have gravitated toward integrated first-trimester algorithms that incorporate maternal history with biophysical indices, such as mean arterial pressure and uterine artery pulsatility, along with circulating placental growth factor (PlGF) [8]. Although such approaches successfully identify approximately 82% of preterm PE cases at a 10% false-positive threshold, their performance in the late onset variant remains notably suboptimal [9]. Moving into the later trimesters, the sFlt-1/PlGF ratio has gained clinical traction, although its utility is largely confined to serving as a robust rule-out tool for women who are already presenting symptomatic [10]. Although progress has been made, a dependable biomarker for the second trimester to identify women at risk of PE before the appearance of symptoms has not yet been developed, limiting the potential for enhanced monitoring and timely medical intervention [11].

Growth arrest-specific protein 6 (Gas6) is a vitamin K-dependent ligand for the TAM receptor tyrosine kinase family (Tyro3, AXL, and MerTK) [12]. Through this receptor axis, Gas6 participates in cell survival, proliferation, migration, immune regulation, and efferocytosis [13]. Within the placenta, Gas6/AXL signaling is implicated in trophoblast invasion and placental vascular remodeling, and dysregulation of this axis contributes to preeclampsia [14]. Transcriptomic analyses have shown that this pathway influences mitochondrial function, extracellular matrix remodelling, and collagen biosynthesis in placental tissue [15]. Disruption of this axis leads to hindered trophoblast invasion, increased oxidative stress, and heightened inflammatory signaling, all of which are well-documented changes in the pathogenesis of PE [16]. Circulating Gas6 concentrations are altered in women with established PE; however, whether these changes are detectable earlier in gestation, and whether they have predictive value, has not been examined [17].

The aim of this study was to determine whether maternal serum Gas6 concentrations measured during routine first-trimester screening (11 + 0 to 13 + 6 weeks) are reduced in women who subsequently develop PE, and to evaluate the predictive value of Gas6 for both early and late onset phenotypes.

## 2. Materials and Methods

### 2.1. Study Design and Setting

This was a single-center, prospective, nested case–control study conducted within a cohort of pregnant women who attended routine first-trimester combined screening at 11 + 0 to 13 + 6 weeks of gestation at the Department of Obstetrics and Gynecology, Samsun Training and Research Hospital. The exposure of interest, maternal serum growth arrest-specific protein 6 (Gas6), was measured in residual first-trimester serum samples. Participants were followed prospectively until delivery, and the diagnosis of preeclampsia, together with gestational age at onset, was recorded from routine clinical records according to ISSHP criteria. Because Gas6 was assayed only after pregnancy outcomes were known and was never available to the clinical team, clinical diagnosis and outcome classification were independent of the biomarker results. This study was designed, conducted, and reported in accordance with the Strengthening the Reporting of Observational Studies in Epidemiology (STROBE) statement for case–control studies.

### 2.2. Ethical Approval and Informed Consent

The study protocol was approved by the Samsun University Faculty of Medicine Non-Interventional Clinical Research Ethics Committee on 20 August 2025 (approval number GOKAEK-2025/17/16), and the study was conducted in accordance with the principles of the Declaration of Helsinki. This was an independent study and not a continuation or secondary analysis of previously approved protocols. At the time of routine first-trimester screening, eligible women were informed about the purpose, procedures, and voluntary nature of the study by a member of the research team, and written informed consent was obtained prior to enrollment. It was explained to every woman that participation was voluntary and that the decision to participate or decline would not affect her clinical care in any way. The consent specifically covered the storage of residual serum, the linkage of these samples to routinely collected clinical data through a coded study identifier, and their use in biomarker analysis. Written informed consent was obtained from all women who entered the prospective cohort, from whom the final case and control groups were subsequently drawn, and not from the analyzed subset alone.

### 2.3. Study Period, Recruitment, and Follow-Up

Recruitment began on 25 August 2025, immediately after ethics approval, and consecutive eligible women presenting for routine first-trimester combined screening were enrolled. Because serum was obtained at 11 + 0 to 13 + 6 weeks and the outcome was ascertained at delivery, the interval between sampling and delivery for an individual participant ranged from approximately 10 weeks for the earliest preterm births at 24 weeks to approximately 28 weeks for births at 39 weeks. To ensure that every enrolled participant could be followed to delivery before the database was locked, including pregnancies that continued to term, recruitment was closed on 26 October 2025, which was 28 weeks before the final delivery date. Enrolled women were then followed prospectively until delivery. The earliest deliveries occurred from early November 2025, and the last delivery in the cohort took place on 10 May 2026. The database was locked after the last delivery. This schedule provided complete outcome ascertainment for the entire cohort and allowed both early onset preeclampsia, defined as a diagnosis before 34 weeks of gestation, and late onset preeclampsia, defined as a diagnosis at or after 34 weeks, to be captured across the entire sample.

### 2.4. Participant Flow and Eligibility

**Figure 1:**
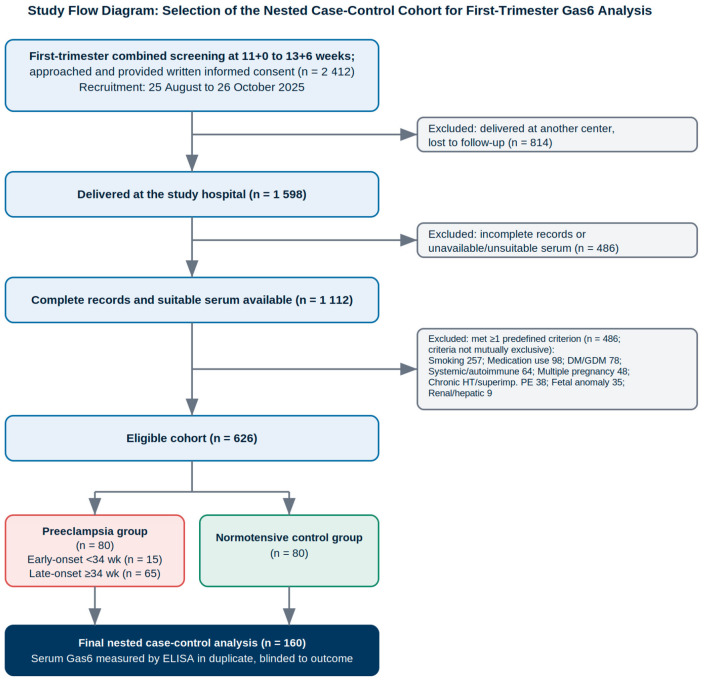
Revised study flow diagram of participant selection and allocation for nested case–control analysis. Abbreviations: DM, diabetes mellitus; GDM, gestational diabetes mellitus; HT, hypertension; PE, preeclampsia. This figure was created using Python (version 3.11).

During the recruitment period, 2412 women who attended routine first-trimester combined screening at 11 + 0 to 13 + 6 weeks were approached and provided written, informed consent. Of these, 814 were subsequently lost to follow-up because they delivered at another center, which prevented complete ascertainment of delivery and neonatal records, leaving 1598 women who delivered at the study hospital. A further 486 women were excluded because of incomplete medical records or unavailable or unsuitable serum samples, leaving 1112 women with complete records and suitable serum samples. The predefined exclusion criteria were applied, and 486 women were excluded. Because these criteria were not mutually exclusive, a single woman could meet more than one criterion; therefore, the sum of individual reasons was greater than the number of excluded women: smoking (*n* = 257), regular medication use other than routine pregnancy supplementation (*n* = 98), pre-existing or gestational diabetes mellitus (*n* = 78), systemic inflammatory or autoimmune disease (*n* = 64), multiple pregnancy (*n* = 48), chronic hypertension or superimposed preeclampsia (*n* = 38), major fetal structural or chromosomal anomaly (*n* = 35), and chronic renal or hepatic disease (*n* = 9). The remaining 626 women formed the eligible cohort from which the cases and controls were selected (Figure 1).

Eligible women had a singleton pregnancy with gestational age confirmed by first-trimester ultrasonography, attended routine first-trimester combined screening between 11 + 0 and 13 + 6 weeks, had residual first-trimester serum available, delivered at the study center, had complete obstetric and neonatal follow-up, and provided informed written consent. Women were excluded if they had a multiple pregnancy, a major fetal structural or chromosomal anomaly, chronic hypertension, superimposed preeclampsia, pre-existing or gestational diabetes mellitus, chronic renal or hepatic disease, systemic inflammatory or autoimmune disease, or a smoking history. Women who used regular medications other than routine pregnancy supplementation, had incomplete records, delivered elsewhere, or whose serum was unavailable or unsuitable were also excluded.

### 2.5. Selection of Cases and Controls

Within the eligible cohort of 626 pregnant women, those who developed preeclampsia during prospective follow-up and met the eligibility criteria were consecutively assigned to the case group, resulting in 80 cases. An equal number of normotensive women who completed pregnancy without preeclampsia and had suitable residual serum samples were consecutively selected from the same cohort as the controls. Controls were drawn from the same source population and recruitment window as the cases, so that the timing of sampling, storage conditions, and assay batches were comparable between the two groups. Cases and controls were not individually matched for maternal characteristics. Maternal age and body mass index were addressed by multivariable adjustment in the analysis. The final nested case–control sample comprised 160 pregnant women, 80 with preeclampsia and 80 normotensive. The preeclampsia group was further classified by timing of onset into early onset preeclampsia (*n* = 15) and late onset preeclampsia (*n* = 65) (Figure 1).

### 2.6. Diagnosis of Preeclampsia

Preeclampsia was diagnosed by the attending obstetricians as part of routine clinical care, according to the 2021 International Society for the Study of Hypertension in Pregnancy criteria [18] and independently of the study because Gas6 was measured later from banked serum and was never available to the clinical team. Preeclampsia was defined as new onset hypertension after 20 weeks of gestation, with a systolic blood pressure of at least 140 mmHg and/or diastolic blood pressure of at least 90 mmHg recorded on two occasions at least 4 h apart, together with proteinuria and/or evidence of maternal organ dysfunction. Blood pressure was measured according to the standard antenatal protocol of the department, and proteinuria was defined as a spot urine protein-to-creatinine ratio of at least 30 mg/mmol or at least 300 mg in a 24 h urine collection. Early onset preeclampsia was defined as a diagnosis before 34 weeks and late onset preeclampsia was defined as a diagnosis at or after 34 weeks. The recorded diagnoses and the gestational age at onset were extracted from electronic medical records by the research team. No biomarker, including Gas6, was used to establish or support the clinical diagnosis.

### 2.7. Sample Size

The minimum sample size was determined a priori for the comparison of maternal serum Gas6 levels between the two independent groups. To detect a large between-group difference corresponding to an effect size of Cohen d = 0.67, with a two-sided significance level of 0.05 and a statistical power of 80%, at least 35 participants were required in each group. The sample size calculation was performed using GPower (version 3.1.9.7). To allow for losses to follow-up and for unavailable or unsuitable serum, and to improve the precision of the estimates, all eligible women who developed preeclampsia during follow-up were included as cases, and an equal number of normotensive controls were consecutively selected from the same eligible cohort, producing a final sample of 80 cases and 80 controls and providing a power well above 80% for the observed differences.

### 2.8. Serum Sampling, Storage, and Gas6 Measurement

Venous blood collected during routine first-trimester combined screening at 11 + 0 to 13 + 6 weeks was used for the analysis, and no additional blood draw or interventional procedure was performed for this study. Within 30 min of collection, the samples were centrifuged at 3000 rpm for 15 min, and the residual serum from women who provided consent was aliquoted and stored at minus 80 °C. Because the nested design required the outcomes to be known before the assay, the samples were kept frozen until batch analysis was performed after the cohort had completed follow-up, which corresponds to a storage duration of approximately 7 to 9 months. Gas6 was measured only in the 160 women who formed the nested case–control sample, and not in the entire screened population.

Serum Gas6 was quantified using a commercial human Gas6 sandwich enzyme-linked immunosorbent assay kit (catalog no. E3257Hu; Bioassay Technology Laboratory [BT Lab], Shanghai, China) in accordance with the manufacturer’s instructions. Concentrations were interpolated from a standard curve prepared from the kit calibrators, and all measured values were within the assay range. Each sample, standard, and control provided with the kit was assayed in duplicate, and the mean of the duplicate readings was used. After the enzymatic reaction, the optical density was read at 450 nm, and any sample exceeding the upper limit of the standard curve was re-assayed after dilution. All assays were performed by laboratory personnel who were blinded to the clinical outcomes and group allocation. In accordance with the manufacturer’s specifications, the intra-assay and inter-assay coefficients of variation were below 8% and below 10%, respectively.

### 2.9. Data Collection

Maternal demographic characteristics, obstetric history, first-trimester screening data, blood pressure measurements, follow-up records, diagnosis and timing of preeclampsia, gestational age at delivery, mode of delivery, neonatal outcomes, Apgar scores, and neonatal intensive care unit admission were retrieved from the institutional electronic medical records using a coded study identifier. All data were reviewed and validated prior to statistical analysis. The participant flow diagram (Figure 1) was prepared using Python (CairoSVG), which is declared here in accordance with journal requirements.

### 2.10. Statistical Analysis

Statistical analyses were performed using IBM SPSS Statistics for Windows (version 26.0; IBM Corp., Armonk, NY, USA).

The distribution of continuous variables was assessed using the Shapiro–Wilk test. Continuous variables were expressed as mean ± standard deviation for normally distributed data and as median with minimum–maximum values for non-normally distributed data. Categorical variables were expressed as frequencies and percentages.

Comparisons between two independent groups were performed using the independent samples *t*-test or the Mann–Whitney U test, as appropriate. Comparisons among more than two groups were performed using one-way analysis of variance or the Kruskal–Wallis test, depending on the data distribution. When a significant difference was detected, post hoc pairwise comparisons were performed with appropriate corrections for multiple testing. Categorical variables were compared using the chi-square test or Fisher’s exact test, as appropriate.

Receiver operating characteristic (ROC) curve analysis was performed to evaluate the diagnostic performance of maternal serum Gas6 levels in predicting preeclampsia, particularly early onset preeclampsia. The area under the curve with 95% confidence intervals was calculated. The optimal cut-off value was selected retrospectively using the Youden index, and the corresponding sensitivity and specificity values were reported as exploratory estimates.

Multivariable logistic regression analysis was performed to assess the association between serum Gas6 levels and early onset preeclampsia after adjustment for maternal age and body mass index. Maternal age and BMI were entered into the model as continuous variables and were not dichotomized. The results were expressed as odds ratios with 95% confidence intervals. Because the number of early onset preeclampsia cases was limited, no additional covariates were added to the model in order to avoid overfitting.

Spearman’s correlation analysis was used to evaluate the association between serum Gas6 levels and the clinical variables. Statistical significance was set at *p* < 0.05.

During the preparation of this manuscript, the authors used GenAI, a generative artificial intelligence tool, only to assist with English language editing and the preparation/layout of the study flow diagram (Figure 1). All AI-assisted output was critically reviewed, edited, and approved by the authors, who take full responsibility for the accuracy and integrity of the content of this publication.

## 3. Results

### 3.1. Study Population and Group Characteristics

A total of 160 pregnant women were included in the final analyses. Based on clinical outcomes and the diagnostic criteria of the International Society for the Study of Hypertension in Pregnancy, participants were classified into three groups: the control group (*n* = 80), early onset preeclampsia group, defined as diagnosis before 34 weeks of gestation (*n* = 15), and late onset preeclampsia group, defined as diagnosis at or after 34 weeks of gestation (*n* = 65). All serum samples for Gas6 quantification were obtained between 11 and 14 weeks of gestation.

### 3.2. Demographic and Clinical Characteristics

The demographic and clinical characteristics of the preeclampsia and control groups are summarized in Table 1. Comparisons between the two groups were performed using the Mann–Whitney U test. The results revealed that age was significantly higher in the preeclampsia group than in the control group (*p* < 0.05). Similarly, body mass index (BMI) was significantly higher in the preeclampsia group than in the control group (*p* < 0.001). Gravidity was significantly lower in the preeclampsia group than in the control group (*p* < 0.001). No statistically significant difference was detected between the groups in terms of parity (*p* > 0.05). Gestational age was significantly lower in the preeclampsia group than in the control group (*p* < 0.001). Furthermore, diastolic blood pressure was significantly higher in the preeclampsia group than in the control group (*p* < 0.001).

### 3.3. Obstetric and Neonatal Outcomes

Table 2 summarizes the obstetric and neonatal outcomes of the control and preeclampsia groups. Categorical variables were compared using the chi-square test. The cesarean section (CS) rate was significantly higher in the preeclampsia group than in the control group (*p* = 0.011). No significant difference was observed in the presence of meconium between the groups (*p* = 0.057). Neonatal intensive care unit (NICU) admission was significantly more frequent in the preeclampsia group (*p* = 0.009).

### 3.4. Maternal Serum Gas6 Levels

Serum Gas6 concentrations were significantly lower in women with preeclampsia than in controls (15.73 [IQR: 11.51–31.27] vs. 38.08 [IQR: 27.62–49.14] ng/mL, *p* < 0.001). Apgar scores were also lower in the preeclampsia group; however, these findings were considered secondary neonatal outcome observations.

#### Serum Gas6 Levels Across Preeclampsia Phenotypes

**Figure 2:**
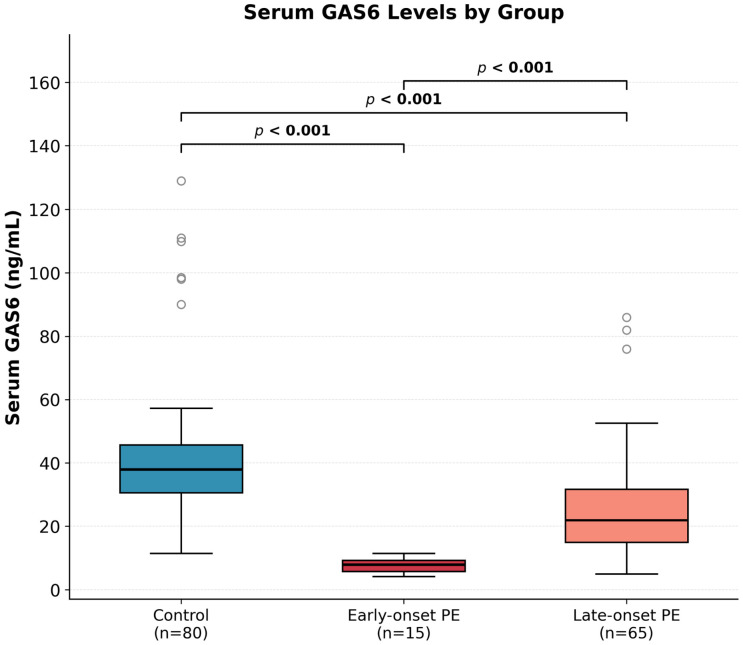
Serum Gas6 concentrations among the control, early onset, and late onset preeclampsia groups, shown as median and interquartile range (Kruskal–Wallis H = 50.61, *p* < 0.001; all pairwise comparisons, *p* < 0.001). Abbreviations: GAS6, growth arrest-specific protein 6; PE, preeclampsia.

Maternal serum GAS6 levels were compared among the early, late onset preeclampsia, and control groups using the Kruskal–Wallis test (Figure 2). A significant overall difference was observed (H = 50.61, *p* < 0.001). Gas6 levels were lowest in the early onset preeclampsia group (6.45 [IQR 5.96–10.25] ng/mL), followed by the late onset group (20.07 [IQR 12.47–37.56] ng/mL), and were highest in the control group (38.08 [IQR 27.62–49.14] ng/mL). Post hoc pairwise comparisons with Bonferroni correction confirmed that all between-group differences were significant (all *p* < 0.001).

### 3.5. Receiver Operating Characteristic (ROC) Analysis

**Figure 3:**
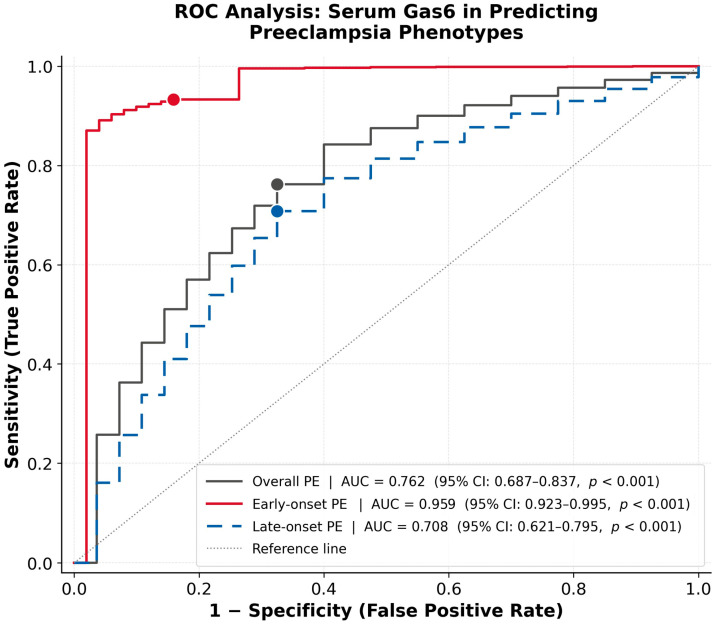
Discriminative performance of first-trimester serum Gas6 levels for preeclampsia phenotypes. Receiver operating characteristic (ROC) curves show the discriminative performance of Gas6 for early onset preeclampsia (solid red line; AUC = 0.959), late onset preeclampsia (dashed blue line; AUC = 0.708), and all preeclampsia cases combined (solid gray line; AUC = 0.762). Markers indicate the optimal cutoff values determined using the Youden index. The diagonal reference line represents a non-discriminatory test (AUC = 0.500). First-trimester serum Gas6 levels showed the highest discriminative performance for early onset preeclampsia.

The predictive value of first-trimester maternal serum GAS6 levels exhibited a clear divergence across the preeclampsia phenotypes (Table 3, Figure 3). For early onset preeclampsia, GAS6 levels demonstrated a high discriminatory capacity, with an AUC of 0.959 (95% CI: 0.923–0.995, *p* < 0.001). At the retrospectively selected threshold of ≤13.82 ng/mL, determined by the Youden index, Gas6 showed a sensitivity of 93.3%, and specificity of 84.1%. Because this cut-off was derived retrospectively from the present dataset, it should be regarded as exploratory and requires external validation. Conversely, the discriminatory value of GAS6 was notably more modest for the late onset phenotype (AUC = 0.708, 95% CI: 0.621–0.795, *p* < 0.001), reflecting the comparatively lower degree of placental dysfunction in late-presenting disease. Although the overall discriminatory performance across all preeclampsia cases was statistically significant (AUC = 0.762, 95% CI: 0.687–0.837, *p* < 0.001), these findings collectively indicate that GAS6 depletion in early pregnancy is most strongly associated with the future development of severe, early onset disease.

### 3.6. Logistic Regression Analysis for Early Onset Preeclampsia

Multivariable logistic regression analysis was performed to assess the association between first-trimester serum Gas6 levels and early onset preeclampsia after adjustment for maternal age and BMI (Table 4). Lower Gas6 levels remained significantly associated with early onset preeclampsia (OR = 0.555, 95% CI: 0.412–0.748, *p* < 0.001). Maternal age (OR = 1.039, 95% CI: 0.801–1.347, *p* = 0.773) and BMI (OR = 1.283, 95% CI: 0.944–1.742, *p* = 0.111) were not statistically significant in this model.

## 4. Discussion

The present study demonstrates that first-trimester serum Gas6 levels are significantly reduced in women who subsequently develop preeclampsia, with a progressive and phenotype-dependent gradient of depletion that is most pronounced in the early onset subgroup. These findings extend the current understanding of Gas6/TAM receptor biology in placentation and provide early clinical evidence that circulating Gas6 levels measured during routine first-trimester screening may have predictive value for preeclampsia, particularly for the early onset phenotype, while remaining a candidate marker that requires confirmation.

The biological plausibility of our findings is robustly supported by the well-established role of Gas6 in maintaining the placental homeostasis. Gas6 serves as the primary ligand for the TAM receptor tyrosine kinase family-Tyro3, AXL, and MerTK-and through this pathway, it exerts anti-apoptotic, anti-inflammatory, and pro-migratory effects on trophoblast cells [12,13]. Gas6/AXL signaling is implicated in trophoblast invasion and placental vascular function, and its dysregulation contributes to defective placentation [15]. In a rat model, Gas6/AXL signaling induced a preeclampsia-like phenotype with impaired trophoblast invasion, placental oxidative stress, and hypertension, and AXL inhibition prevented disease progression [15,16]. Stepan et al. reported increased serum Gas6 in established preeclampsia, in contrast to the lower first-trimester concentrations observed here, which indicates that the direction of change varies with gestational timing, assay, and population [19]. In a recent study, Jackson et al. demonstrated through transcriptomic analysis that Gas6-induced placental dysfunction is characterized by extensive disruption of extracellular matrix remodeling, interleukin signaling, and oxidative stress pathway [15]. Inhibition of AXL reversed these transcriptomic changes and restored mitochondrial function, underscoring the central role of this axis [20]. Increased placental TYRO3 and GAS6 mRNA expression has also been reported in women with preeclampsia, suggesting local dysregulation of the TAM receptor system at the maternal–fetal interface [21].

Within this framework, the notably lower first-trimester Gas6 concentrations observed in women who later developed PE align with a model suggesting that early depletion of circulating Gas6, or its increased consumption due to dysregulated TAM receptor signaling, indicates a pre-clinical state of impaired trophoblast invasion and placental vascular dysfunction. The finding that Gas6 concentrations were approximately six times lower in early onset PE than in controls, and three times lower than in the late onset subgroup, is particularly noteworthy. Early onset PE is characterized by a more severe, predominantly placenta-driven pathophysiology stemming from defective deep placentation. In contrast, late onset disease is more significantly influenced by maternal constitutional factors, such as obesity, metabolic syndrome, and pre-existing endothelial dysfunction [7,22]. The steeper Gas6 gradient in early onset PE aligns with a greater degree of TAM receptor axis disruption, indicative of more profound trophoblast insufficiency.

ROC analysis indicated an AUC of 0.959 for early onset preeclampsia, with a sensitivity of 93.3% and a specificity of 84.1% at ≤13.82 ng/mL. However, this estimate was derived from only 15 early onset cases in a single center and was not externally validated; therefore, the ROC-derived cutoff and regression estimates should be regarded as exploratory and hypothesis-generating rather than confirmatory because small case numbers can inflate apparent discrimination and widen uncertainty. For context, first-trimester placental growth factor (PlGF), one of the most widely studied angiogenic biomarkers for PE screening, has shown AUC values of approximately 0.71 for overall PE and 0.80 for early onset PE in a recent nested case–control study [23]. In contrast, the sFlt-1/PlGF ratio has primarily been validated as a short-term rule-out tool in women with suspected PE rather than as a first-trimester screening biomarker [10]. Any comparison with these established markers should be made cautiously, given the differences in design, gestational age at sampling, population, and sample size, and the integration of Gas6 into existing algorithms would require validation in larger independent cohorts.

The discriminatory performance for overall and late onset preeclampsia was moderate (AUC 0.762 and 0.708, respectively). Late onset disease is more heterogeneous, with a smaller placental contribution and a greater role for maternal metabolic and cardiovascular factors, which limits the utility of a single first-trimester biomarker [5,7,22]. In contrast, early onset PE is more strongly associated with defective placentation, including impaired spiral artery remodeling and deep placentation abnormalities [6]. Accordingly, the utility of a single first-trimester biomarker may be limited in late onset PE. Furthermore, the larger late onset PE group in the present study (*n* = 65 vs. *n* = 15 for early onset PE) may have introduced greater phenotypic heterogeneity, thereby diluting the diagnostic signal. These findings are consistent with the broader evidence that late onset PE is more difficult to predict using placental biomarkers alone [24,25].

Apgar scores were lower among neonates born to women with preeclampsia, consistent with the expected adverse neonatal profile of this condition. Although Gas6 levels showed an association with Apgar scores in exploratory analysis, this finding was secondary and should be interpreted cautiously. Dedicated studies would be required to determine whether Gas6 is directly related to neonatal adaptation or whether this association reflects the underlying severity of placental disease. In multivariable logistic regression, lower first-trimester serum Gas6 levels remained significantly associated with early onset PE after adjustment for maternal age and BMI (OR = 0.555, 95% CI: 0.412–0.748, *p* < 0.001). Age and BMI were analyzed as continuous variables. Given the limited number of early onset cases, this model was intentionally restricted to avoid overfitting. Although maternal BMI is a well-established risk factor for PE [2,7], it did not achieve independent significance in this model (*p* = 0.111), despite its significant univariate association (*p* = 0.007). This may reflect the fact that BMI primarily predisposes women to late onset PE through maternal metabolic and cardiovascular pathways [4,5], whereas early onset disease is more strongly driven by placental mechanisms a dimension captured by Gas6 [6]. The independence of Gas6 association from maternal age and BMI underscores its potential as a biomarker, providing complementary information for clinical risk stratification.

### Limitations

The present study has several limitations. First, the early onset preeclampsia subgroup included only 15 cases, reflecting the relative rarity of this phenotype. In addition, the ROC-derived cut-off values were selected retrospectively from the present dataset and were not prespecified; therefore, they should not be interpreted as validated clinical thresholds. This limited sample size may have reduced the precision of subgroup estimates and the robustness of multivariable modeling; therefore, the ROC-derived cutoff value and logistic regression findings should be regarded as exploratory and require validation in larger independent cohorts. Second, the single center design may limit the generalizability of the findings to populations with different demographic and clinical characteristics. Third, controls were selected consecutively rather than individually matched to cases for potential confounders, such as parity or BMI, which may have introduced residual confounding. Although multivariable adjustment was performed for selected maternal variables, unmeasured confounding cannot be excluded. Fourth, the exclusion of a substantial number of participants, particularly due to smoking (*n* = 257), may have limited the representativeness of the study population and should be considered when interpreting the results. Finally, the association between circulating Gas6 levels and neonatal Apgar scores should be interpreted cautiously, as the underlying biological mechanisms remain incompletely characterized and require dedicated mechanistic investigation.

## 5. Conclusions

First-trimester maternal serum Gas6 concentrations were significantly and progressively reduced across preeclampsia phenotypes, with the most pronounced reduction in early onset disease, and lower Gas6 remained associated with early onset preeclampsia after adjustment for maternal age and BMI. Taken together, these findings support Gas6 as a biologically plausible candidate biomarker rather than a validated screening test; the high discriminatory performance for early onset disease was based on a small number of cases and requires confirmation. Multicenter, prospective, externally validated studies are needed before any first-trimester screening application can be considered, including assessment of the incremental value of Gas6 within integrated screening algorithms.

## Figures and Tables

**Table 1:** Comparison of demographic and clinical characteristics of the control and preeclampsia groups.

Variable	Control (*n* = 80)	Preeclampsia (*n* = 80)	*p*
Age (years)	26.00 (20–36)	27.50 (23–37)	0.027
BMI (kg/m^2^)	25 (20–30)	27 (21–34)	**<0.001**
Gravidity	2 (1–5)	2(1–5)	**<0.001**
Parity	1 (0–2)	1 (0–4)	0.056
Gestational age at delivery (weeks)	39.00 (36–39)	37.00 (24–39)	**<0.001**
Diastolic BP (mmHg)	62.50 (50–80)	100.00 (90–120)	**<0.001**

Data are presented as median (min–max). Comparisons between the two groups were performed using the Mann–Whitney U test. Bold *p*-values indicate statistical significance (*p* < 0.05). BMI: body mass index; BP: blood pressure. Blood pressure values were extracted from routine clinical records as documented in the medical files.

**Table 2:** Comparison of maternal serum Gas6 levels and obstetric and neonatal outcomes between control and preeclampsia groups.

Variable		Control (*n* = 80)	Preeclampsia (*n* = 80)	*p*
Mode of Delivery (Normal/CS)	Vaginal Delivery	54 (67.5)	38 (47.5)	0.011
	Cesarean Section	26 (32.5)	42 (52.5)	
Meconium	Absent	71 (88.8)	62 (77.5)	0.057
	Present	9 (11.3)	18 (22.5)	
NICU	Absent	71 (88.8)	58 (72.5)	0.009
	Present	9 (11.3)	22 (27.5)	
Gas6 (ng/mL)		38.08 (27.62–49.14)	15.73 (11.51–31.27)	<0.001
1 min Apgar score		9 (9–9)	7 (7–9)	<0.001
5 min Apgar score		10 (9–10)	8 (7–9)	<0.001

Categorical variables are presented as *n* (%), and continuous or ordinal variables are presented as median (interquartile range). Categorical variables were compared using chi-square tests, and continuous or ordinal variables were compared using the Mann–Whitney U test. Apgar scores are presented as integer values. CS: cesarean section; NICU: neonatal intensive care unit.

**Table 3:** ROC analysis results of serum Gas6 (ng/mL) levels in preeclampsia phenotypes.

Phenotype	Overall PE (*n* = 80)	Early Onset PE (*n* = 15)	Late Onset PE (*n* = 65)
AUC (95% CI)	0.762 (0.687–0.837)	0.959 (0.923–0.995)	0.708 (0.621–0.795)
*p*-value	<0.001	<0.001	<0.001
Optimal cutoff (ng/mL)	≤31.41	≤13.82	≤31.41
Sensitivity (%)	76.2	93.3	70.8
Specificity (%)	67.5	84.1	67.5

Data represent ROC curve analyses comparing each preeclampsia phenotype with that of the controls. The area under the curve (AUC) is presented with 95% confidence intervals (CIs). The optimal cut-off value was selected retrospectively using the Youden index. Gas6: growth arrest-specific protein 6; AUC: area under the curve; CI: confidence interval; PE: preeclampsia.

**Table 4:** Multivariable logistic regression analysis for early onset preeclampsia.

Variables	OR (%95 CI)	*p*
GAS6 (ng/mL)	0.555 (0.412–0.748)	<0.001
Age, years	1.039 (0.801–1.347)	0.773
BMI, kg/m^2^	1.283 (0.944–1.742)	0.111

The multivariable model included Gas6, maternal age, and BMI. Maternal age and BMI were entered as continuous variables and were not dichotomized. Odds ratios represent the change in odds per one-unit increase in each variable. Abbreviations: BMI, body mass index; CI, confidence interval; GAS6, growth arrest-specific protein 6; OR, odds ratio.

## Data Availability

The data presented in this study are available on request from the corresponding author.

## Abbreviations

The following abbreviations are used in this manuscript:

AUC	Area under the curve
AXL	AXL receptor tyrosine kinase
BMI	Body mass index
CI	Confidence interval
ELISA	Enzyme-linked immunosorbent assay
Gas6	Growth arrest-specific protein 6
IQR	Interquartile range
MerTK	Mer tyrosine kinase
NICU	Neonatal intensive care unit
OR	Odds ratio
PE	Preeclampsia
PlGF	Placental growth factor
ROC	Receiver operating characteristic
sFlt-1	Soluble fms-like tyrosine kinase-1
TAM	Tyro3, AXL, MerTK receptor family
Tyro3	Tyrosine-protein kinase receptor 3
